# The Feature Ambiguity Mitigate Operator model helps improve bone fracture detection on X-ray radiograph

**DOI:** 10.1038/s41598-021-81236-1

**Published:** 2021-01-15

**Authors:** Hui-Zhao Wu, Li-Feng Yan, Xiao-Qing Liu, Yi-Zhou Yu, Zuo-Jun Geng, Wen-Juan Wu, Chun-Qing Han, Yong-Qin Guo, Bu-Lang Gao

**Affiliations:** 1 Department of Radiology, Third Hospital of Hebei Medical University, Hebei Province, Shijiazhuang, 050000 People’s Republic of China; 2 DeepWise AI Lab, Beijing, People’s Republic of China; 3 Department of Radiology, Second Hospital of Hebei Medical University, Hebei Province, Shijiazhuang, 050000 People’s Republic of China

**Keywords:** Computational biology and bioinformatics, Diseases, Health care, Medical research, Engineering

## Abstract

This study was performed to propose a method, the Feature Ambiguity Mitigate Operator (FAMO) model, to mitigate feature ambiguity in bone fracture detection on radiographs of various body parts. A total of 9040 radiographic studies were extracted. These images were classified into several body part types including 1651 hand, 1302 wrist, 406 elbow, 696 shoulder, 1580 pelvic, 948 knee, 1180 ankle, and 1277 foot images. Instance segmentation was annotated by radiologists. The ResNext-101+FPN was employed as the baseline network structure and the FAMO model for processing. The proposed FAMO model and other ablative models were tested on a test set of 20% total radiographs in a balanced body part distribution. To the per-fracture extent, an AP (average precision) analysis was performed. For per-image and per-case, the sensitivity, specificity, and AUC (area under the receiver operating characteristic curve) were analyzed. At the per-fracture level, the controlled experiment set the baseline AP to 76.8% (95% CI: 76.1%, 77.4%), and the major experiment using FAMO as a preprocessor improved the AP to 77.4% (95% CI: 76.6%, 78.2%). At the per-image level, the sensitivity, specificity, and AUC were 61.9% (95% CI: 58.7%, 65.0%), 91.5% (95% CI: 89.5%, 93.3%), and 74.9% (95% CI: 74.1%, 75.7%), respectively, for the controlled experiment, and 64.5% (95% CI: 61.3%, 67.5%), 92.9% (95% CI: 91.0%, 94.5%), and 77.5% (95% CI: 76.5%, 78.5%), respectively, for the experiment with FAMO. At the per-case level, the sensitivity, specificity, and AUC were 74.9% (95% CI: 70.6%, 78.7%), 91.7%% (95% CI: 88.8%, 93.9%), and 85.7% (95% CI: 84.8%, 86.5%), respectively, for the controlled experiment, and 77.5% (95% CI: 73.3%, 81.1%), 93.4% (95% CI: 90.7%, 95.4%), and 86.5% (95% CI: 85.6%, 87.4%), respectively, for the experiment with FAMO. In conclusion, in bone fracture detection, FAMO is an effective preprocessor to enhance model performance by mitigating feature ambiguity in the network.

## Introduction

Orthopedic trauma or fracture is an important part of clinical epidemics^1,2^. X-ray examination is the primary and initial measure in radiographic examinations for fracture patients, and fractures can be diagnosed with certainty in most cases in this way. Although X-ray radiographs only provide limited information due to limited projection views and clinic observations, they still yield crucial evidence for triage determination. Accuracy and instantaneity of the report on the radiograph is necessary to provide patients with proper treatment. With the rapid development of artificial intelligence (AI), researchers have begun to dig in employing deep learning AI to improve both the speed and accuracy of X-ray diagnoses of fractures. Most researchers use classification or object detection pipelines of the AI technique to detect fractures in radiographs. To do this, most of the authors actuate deep learning models to work in a binary classification task, i.e., classifying the radiographs into two groups of “fracture” and “non-fracture”. However, this method usually applies a large quantity of data for analysis. Olczak et al.^3^ extracted 256,458 hand, wrist, and ankle radiographs and marked them with classification labels. In their dataset, the VGG16 network achieved an 83% accuracy. The limitation of this study was that the images had to be resized to 256 × 256 resolution (1/10 of the original size), which could make small fractures undetectable. This problem was fixed by Gale et al.^4^ by processing the image through cropping and resizing a region around the neck of the femur (area of interest) to 1024 × 1024 pixels. Huang et al.^5^used a densely-connected convolutional neural network (CNN) structure, the DenseNet, and achieved an AUC (area under the curve) of 99.4%, which indicates that with appropriate architecture and massive amounts of data on specific body parts, deep learning models can be extremely precise. Despite the astonishing performance, the model was limited to the frontal view, neglecting those fractures hidden in other views. Moreover, large amounts of data have always been required, and hence the tremendous cost of time. Kitamura et al.^6^ attempted to minimize the training set data while maintaining the ability of the detecting system. In their study, they used only 1441 of frontal and lateral ankle images as the training data, employed the de novo training and ensemble technique, and achieved an accuracy of 81%, which was comparable to the Olczak’s 83% accuracy achieved by using a massive data^3^.

Lack of interpretability has always been a denounced aspect of the deep CNN learning methods. Classification pipelines can only judge “fracture” or “non-fracture” in the entire image, thus being less useful. Realizing this, Thian et al.^7^ proposed an object detection pipeline, the Faster-RCNN, to locate the region of fracture on wrist radiographs by “bounding-box”. Trained on around 6780 images, the method hit an AUC of 91.8% in the frontal view and 93.3% in the lateral view on each image level. Lindsey et al.^8^ tried another way, the semantic segmentation, to locate fractures in the target wrist area (ulna and radius) by identifying whether each pixel in the image is fracture pixel or not. In their study, the average sensitivity was 80.8% with an 87.5% specificity on the 135,845 body parts labeled, including 34,990 anteroposterior and lateral wrist views based on the 31,490 images used as the final segmentation (detection) for training.

In previous studies with the AI technique to help diagnose bone fractures, a large quantity of data is needed, and lack of interpretability and imaging ambiguity are also two shortcomings. To overcome these shortcomings, we proposed the Feature Ambiguity Mitigate Operator (FAMO) method to mitigate the feature ambiguity of bone fracture images in this study. Moreover, an object detection pipeline was employed, and two network structures of ResNeXt^9^ and FPN^10^ were updated to set a strong baseline. These measures were to decrease the amount of data used to analyze fractures to about 1000 frontal and lateral X-ray radiographs, and by using far fewer data, our purpose was to test if a good diagnostic performance could be achieved.

## Results

### Experiments

We conducted experiments to verify that the proposed FAMO operator can improve the fracture detection performance.

As shown in Tables 1 and 2, the model with the FAMO operator outperformed the control model (ResNeXt101+FPN) in all evaluation scopes. In the box level or per-fracture level metric, FAMO increased the AP by 0.6% (from 76.8 to 77.4%). For per-image sensitivity and specificity, the improvements were 2.4% (from 61.9 to 64.5%) and 1.4% (from 91.5 to 92.9%), respectively. Also, FAMO gained the per-image level AUC by 2.6% (from 74.9 to 77.5%). For per-case sensitivity and specificity, the improvements were 2.6% (from 74.9 to 77.5%) and 1.7% (from 91.7 to 93.4%), respectively. Finally, FAMO enhanced the per-case level AUC by 0.8% (from 85.7 to 86.5%).

**Table 1:** Improvement of FAMO in per-image and per-fracture level.

Network	Per-image sensitivity	Per-image specificity	Youden Index (%)	AUC (%)	Per-fracture AP (%)
ResNeXt101+FPN	579/935 (61.9%)	801/875 (91.5%)	53.4	74.9	76.8
	CI: (58.7%, 65.0%)	CI: (89.5%, 93.3%)	CI: (48.2, 58.6)	CI: (74.1, 75.7)	CI: (76.1, 77.4)
FAMO+ResNext101+FPN	603/935 (64.5%)	813/875 (92.9%)	57.4	77.5	77.4
	CI: (61.3%, 67.5%)	CI: (91.0%, 94.5%)	CI: (52.3, 62.5)	CI: (76.5, 78.5)	CI: (76.6, 78.2)

Note: ResNeXt101, a network structure; FPN, feature pyramid network; FAMO, Feature Ambiguity Mitigate Operator (FAMO) method; AUC, area under the curve; AP, average precision; CI, confidence interval. All CIs were calculated at the two-tailed significance of 0.05.

**Table 2:** The improvement of FAMO in per-case level.

Network	Per-case sensitivity	Per-case specificity	Youden Index (%)	AUC (%)
ResNeXt101+FPN	349/466 (74.9%)	443/483 (91.7%)	66.6	85.7
	CI: (70.6%, 78.7%)	CI: (88.8%, 93.9%)	CI: (59.4, 73.8)	CI: (84.8, 86.5)
FAMO+ResNext101+FPN	361/466 (77.5%)	451/484 (93.4%)	70.9	86.5
	CI: (73.3%, 81.1%)	CI: (90.7%, 95.4%)	CI: (64.0, 77.8)	CI:(85.6, 87.4)

ResNeXt101, a network structure; FPN, feature pyramid network; FAMO, Feature Ambiguity Mitigate Operator (FAMO) method; AUC, area under the curve; AP, average precision; CI, confidence interval. All CIs were calculated at the two-tailed significance of 0.05.

**Figure 1:**
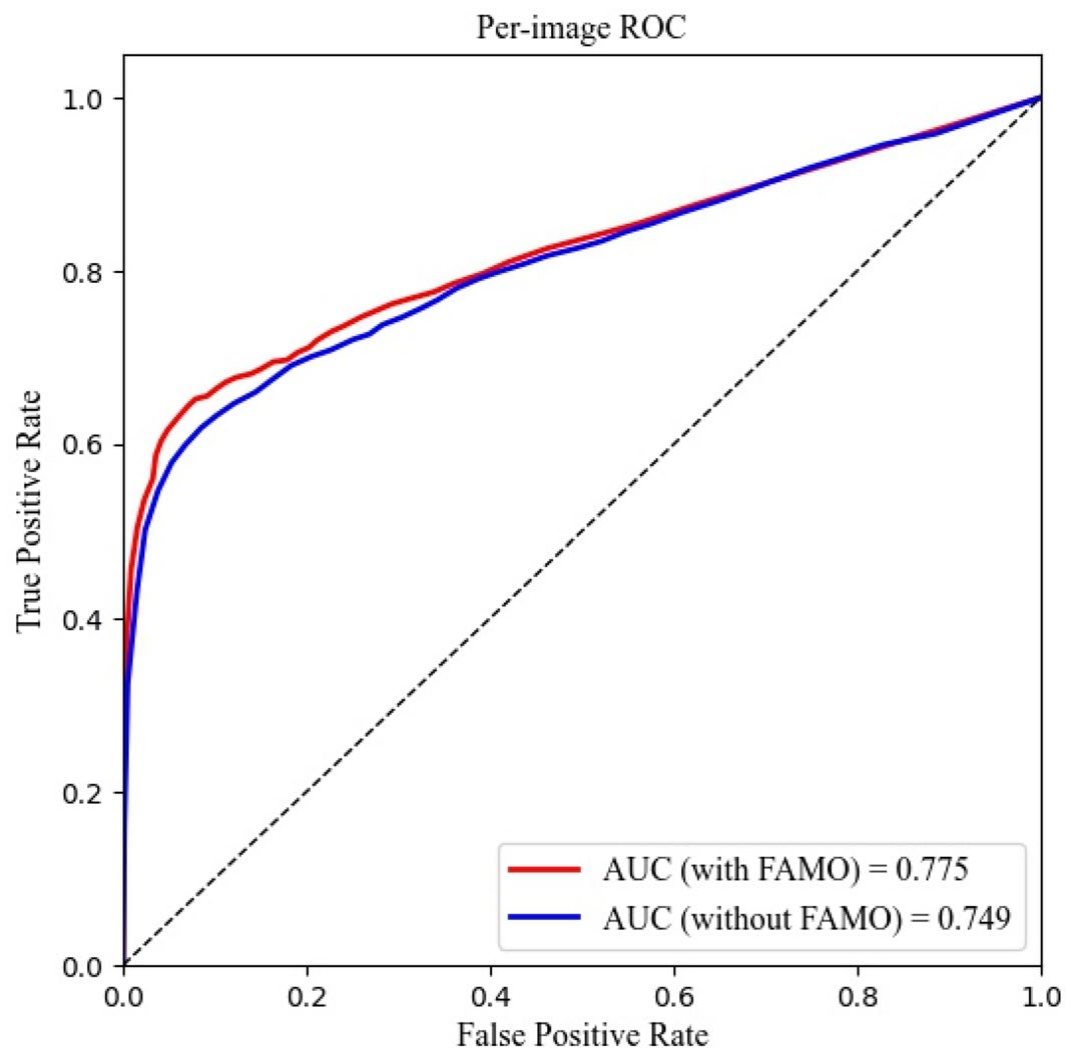
Per-image level ROC curve.

**Figure 2:**
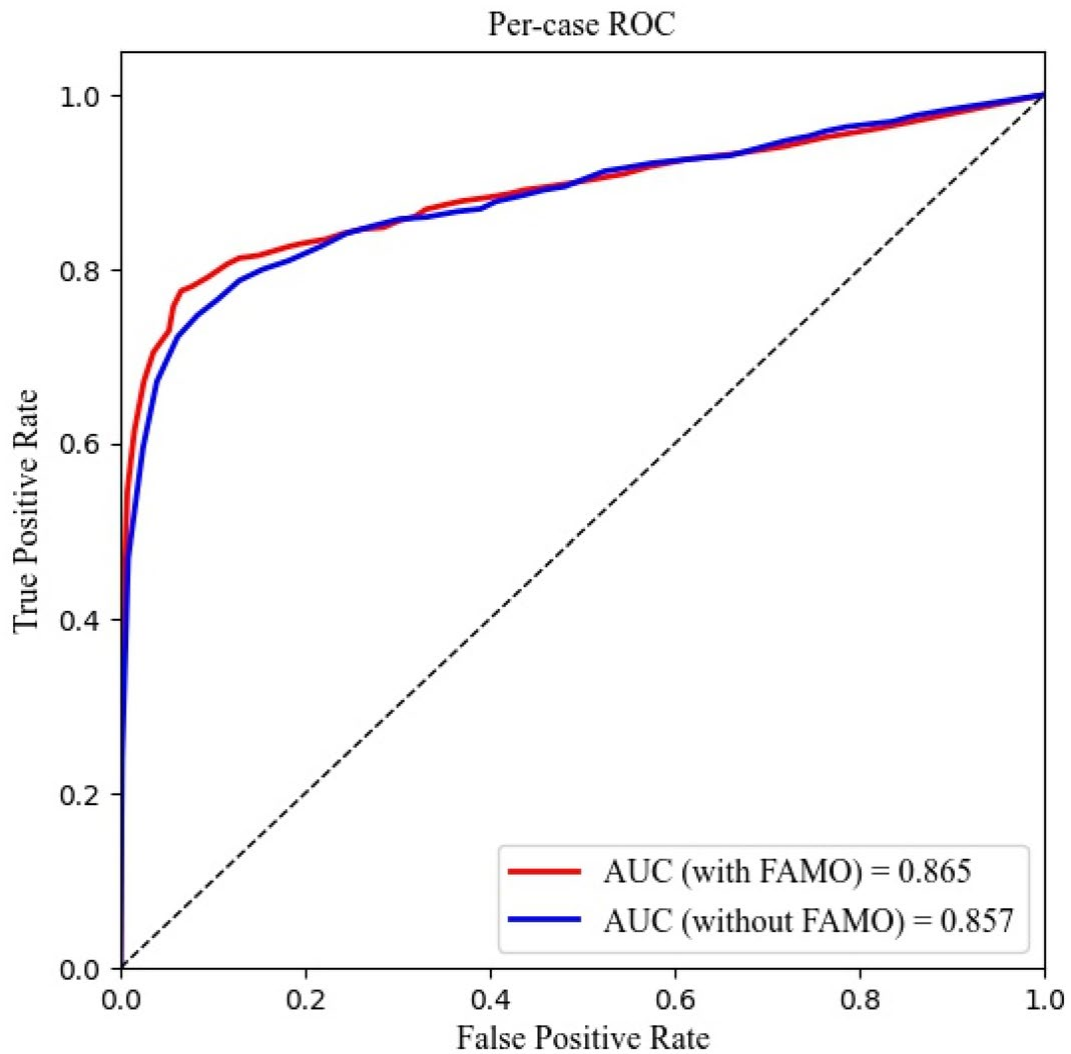
Per-case level ROC curve.

The per-image and per-study ROC curve were illustrated in Figs. 1 and 2, respectively.

We performed hypothesis tests for both per-image and per-case level, which hypothesized that the FAMO performance was worse than the performance in the control group model. The P-values to deny them were shown in the Table 3.

**Table 3:** Hypothesis test results.

Zero hypothesis	*P* value
SEN_FAMO_ < SEN_control_ at per-image level	0.051
SPEC_FAMO_ < SPEC_control_ at per-image level	0.028
SEN_FAMO_ < SEN_control_ at per-case level	0.097
SPEC_FAMO_ < SPEC_control_ at per-case level	0.087

SEN_FAMO_, sensitivity of model with FAMO; SEN_control_, sensitivity of control model; SPEC_FAMO_, specificity of model with FAMO; SPEC_control_, specificity of control model; *P* value, the probability to obtain a result as extreme as that in the hypothesis test which assumed that FAMO performance was worse than the control model.

**Figure 3:**
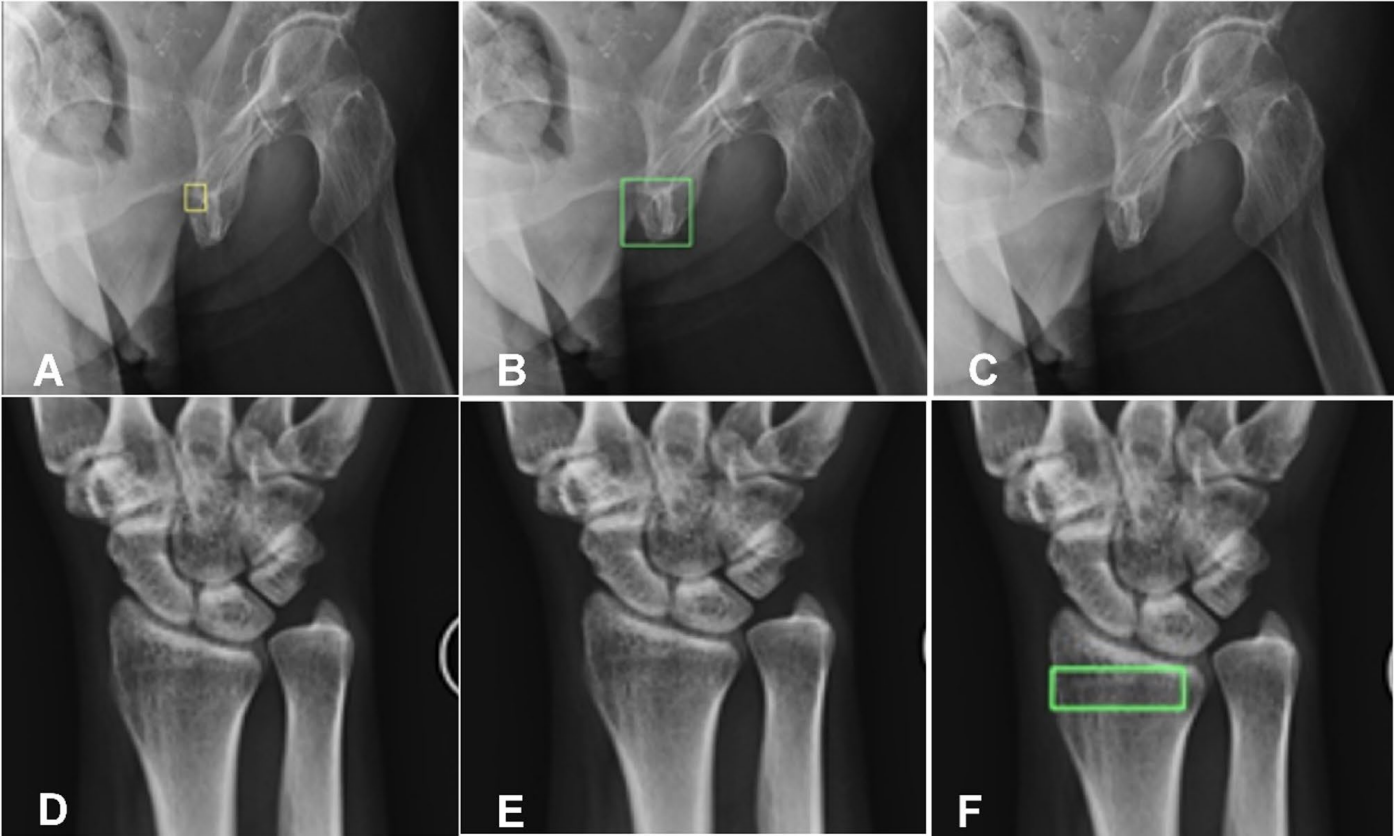
Effects of the FAMO (Feature Ambiguity Mitigate Operator) plus ResNeXt101+FPN model and the ResNeXt101+FPN model only, with the FAMO plus ResNeXt101+FPN model being helpful in finding bone fractures. (**A**) A minor fracture was marked by the radiologist (small box) on the radiograph. (**B**) The fracture line was successfully recognized by the FAMO plus ResNeXt101+FPN model (green box). (**C**) The ResNeXt101+FPN model did not recognize the fracture. No fracture line was detected on the radiograph by the radiologist (**D**) or by the FAMO plus ResNeXt101+FPN model (**E**). But the ResNeXt101+FPN model (causing feature distortion) detected a false positive fracture line (**F**, green box).

**Figure 4:**
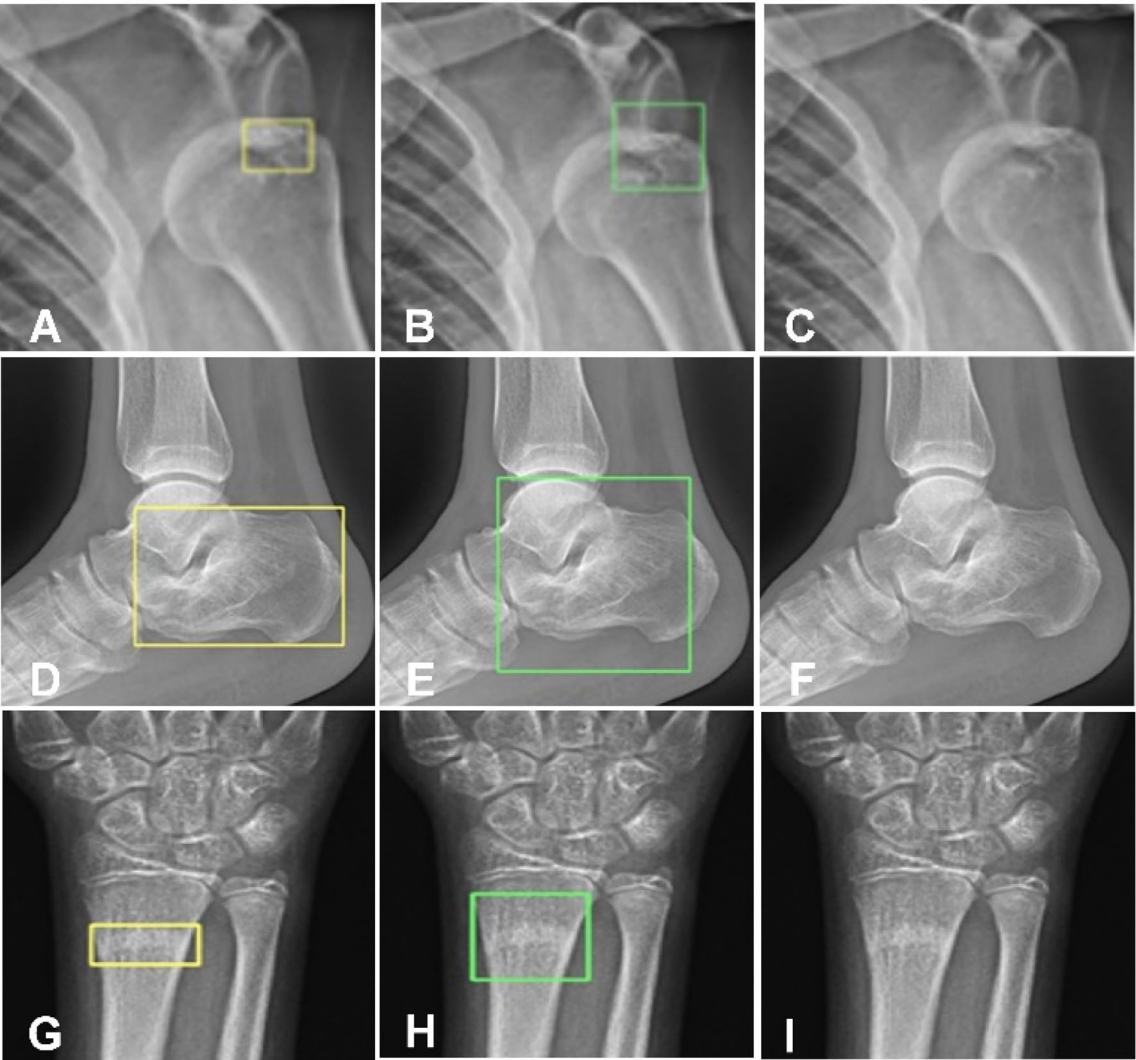
Effects of the Feature Ambiguity Mitigate Operator (FAMO) plus ResNeXt101+FPN model and the ResNeXt101+FPN model only, with the FAMO plus ResNeXt101+FPN model being helpful in finding bone fractures. (**A**–**C**) Dislocation of the left shoulder joint. (**A**) The radiologist correctly located the dislocation of the shoulder joint. (**B**) The FAMO plus ResNeXt101+FPN model also correctly pointed out the location of dislocation. (**C**) The ResNeXt101+FPN model only did not find the dislocation. (**D**–**F**) Compression fracture of the calcaneus. (**D**) The radiologist correctly found the fracture site. (**E**) The FAMO plus ResNeXt101+FPN model also correctly pointed out the fracture area. (**F**) The ResNeXt101+FPN model failed to find the fracture. (**G**–**I**) Radial embedded fracture. (**G**) The radiologist correctly found the embedded fracture. (**H**) The FAMO plus ResNeXt101+FPN model also correctly pointed out the fracture. (**I**) The ResNeXt101+FPN model failed to find the fracture.

In different body-parts (Figs. 3 and 4), the model performance was analyzed regarding the fracture recognizing difficulty relative to the data volume of the body part (Table 4). Among the body parts of elbow, shoulder, and knee with fewer data (Table 5), knee fractures were relatively hard for the model to recognize (with the per-case AUC of 82.4%) while shoulder fractures were more easily recognized (83.2% per-case AUC). Among body parts with relatively sufficient data, pelvic fractures were nearly perfect for the machine to learn, resulting in a 96.9% per-case AUC and a 94.4% per-case sensitivity, which meant all images in the test dataset were recalled. For foot fractures, even with more (1277) radiographs and FAMO operator enhancement, the model only came up with an 82.1% per-case AUC, which might be due to the massive amount of bone blocks in the foot to complicate overlapping between them.

**Table 4:** Body-part specificity and sensitivity data.

Body part	Per-image sensitivity (%)	Per-image specificity (%)	Per-image AUC (%)	Per-image Youden Index (%)	Per-case sensitivity (%)	Per-case specificity (%)	Per-case AUC (%)	Per_image Youden Index (%)
Hand	66.3	92.0	80.7	58.3	70.5	95.0	85.3	65.5
Wrist	77.0	91.8	84.5	68.8	82.0	93.2	86.8	75.2
Elbow	56.2	95.4	75.5	51.6	63.2	91.1	71.1	54.2
Shoulder	64.2	95.2	76.2	59.4	74.7	94.8	83.2	69.5
Pelvic	85.3	94.3	92.5	79.6	94.4	95.8	96.9	90.2
Knee	37.4	92.6	51.9	30.0	72.7	87.1	82.4	59.8
Ankle	60.1	93.7	75.3	53.8	81.7	93.3	88.5	75.0
Foot	67.8	86.7	78.6	54.4	77.6	81.5	82.1	59.1

AUC, area under the curve.

**Table 5:** Data distribution in body parts and different views.

Body part	Anteroposterior view	Other views	Total
Hand	1646	5	1651
Wrist	827	475	1302
Elbow	189	217	406
Shoulder	685	11	696
Pelvic	1572	8	1580
Knee	944	4	948
Ankle	829	351	1180
Foot	711	566	1277
Total	7403	1637	9040

## Discussion

In this study, we presented the FAMO plus ResNeXt101+FPN model to mitigate the feature ambiguity of bone fracture images using far few data to analyze the radiograph images and had reached a diagnostic performance as good as that by radiologists, but better than that by the ResNeXt101+FPN model only. By using 406 to 1651 desensitized radiographs of different body parts, the FAMO plus ResNeXt101+FPN model achieved better sensitivity, specificity, and AUC than the ResNeXt101+FPN model only, and the FAMO plus ResNeXt101+FPN model also obtained a per-case AUC mostly above 80% in different body parts.

In the study of AI for analyzing orthopedic trauma radiographs, Olczak et al.^3^ extracted 256,458 wrist, hand, and ankle radiographs and achieved an accuracy of at least 90% when identifying laterality, body part, and exam view in all networks with the final accuracy for fractures being estimated at 83% for the best performing network. This network performed similarly to senior orthopedic surgeons when presented with images at the same resolution as the network. In the study using CNN for automated fracture detection and localization on wrist radiographs, Thian et al.^7^ used around 6780 images to reach an AUC of 91.8% in the frontal view and 93.3% in the lateral view on each image level. In the study of deep neural network for improving fracture detection, Lindsey et al.^8^ used the semantic segmentation to locate fractures in the target wrist area (ulna and radius) by identifying whether each pixel in the image is fracture pixel or not. In this study, they used 135,409 radiographs to reach a sensitivity of 80.8% (95% CI: 76.7–84.1%) unaided and 91.5% (95% CI: 89.3–92.9%) aided, and a specificity of 87.5% (95% CI: 85.3–89.5%) unaided and 93.9% (95% CI: 92.9–94.9%) aided. The misinterpretation rate was averagely decreased by 47.0% (95% CI: 37.4–53.9%).

The FAMO plus ResNeXt101+FPN model proposed in our study achieved better sensitivity, specificity, and AUC with the per-case AUC mostly above 80% in different body parts. However, there are also some limitations in our study. First, the data were imbalance to some extent, and the amounts of some body parts were far from sufficient for a deep learning system. Second, radiologists had annotated segmentation of fracture areas, which we did not make use of. The minor experiments revealed that a model only supervised by box label rather than segmentation label could have better performance, the cause of which was not investigated in this study. Third, the data for training and testing were from the same institution with no involvement of multiple centers, and the robustness of the model on the data of different distributions from different centers remained to be verified. Future studies will have to resolve these issues for better performance.

In conclusion, the novel operator FAMO proposed in this study is able to mitigate the feature ambiguity in X-ray bone fracture detection, improving both sensitivity and specificity across per-fracture, per-image, and per-case level in all body parts.

## Methods

This study was approved by the institutional review board of the Third Hospital of Hebei Medical University (K2019-036-1), and the study was conducted in accordance with the ethical standards laid down in the 1964 Declaration of Helsinki and its later amendments. Written informed consent was waived due to the retrospective nature of this study. All methods were performed in accordance with the relevant guidelines and regulations. A total of 9040 desensitized radiographs of various body parts and projection views were drawn from the pictures archiving and communication system (PACs) and annotated separately for training, validating and testing. The dataset distribution is shown in Table 5.

### Data labeling

The data were labeled by two radiologists with ten-year’s experience using the segmentation method similar to that by Lindsey et al.^8^. The data labeling was improved in three folds. Firstly, considering bone blocks in perspective X-ray images sometimes stacking over each other, the doctors were required to label an instance segmentation instead of semantic segmentation, which allows areas of different fractures to overlap. Secondly, parts that should be included in a fracture segmentation area were precisely defined, including fracture surface, articular surface collapse, and abnormal bone trabecula. Clarifying fracture symptoms not only eliminated the heterogeneity of labels marked by different doctors, but also helped them stay alert as they kept focusing on delineating fracture areas. Thirdly, all labels were finally carefully reviewed by the chief physician with 20 years of experience. The bounding-boxes used to train and test the models were generated automatically according to each instance segmentation, a process that could harvest more accurate bounding-boxes than direct annotations. The main purpose of this study was to compare the effect of the FAMO model and baseline model, so data labeling by the physician was used as the gold standard. The consistency (credibility) of data labeling by physicians is shown in Table 6, with the excellent agreement achieved in most parts except for the elbow which had fair to good agreement between physicians.

**Table 6:** Reliability of the gold standards for the fracture dataset.

Body part	Cohen’s κ			All Fleiss’ κ
	Between physicians 1 and 2	Between physicians 1 and 3	Between physicians 2 and 3	
Hand	0.95	0.97	0.92	0.94
Wrist	0.74	0.81	0.73	0.76
Elbow	0.83	0.72	0.67	0.74
Shoulder	0.85	0.95	0.79	0.86
Pelvic	0.85	0.87	0.86	0.86
Knee	0.81	0.88	0.79	0.83
Ankle	0.93	0.95	0.92	0.93
Foot	0.83	0.86	0.77	0.82
All	0.85	0.88	0.81	0.85

The guidelines of Fleiss and colleagues characteristic κ values of more than 0.75 were set as excellent agreement, 0.40–0.75 as fair to good agreement, and less than 0.40 as poor agreement beyond chance.

### Pre-processing

Image arrays in 16-bit integer format were extracted from Digital Imaging and Communications in Medicine (DICOM) files. The raw data were processed with adaptive histogram equalization^11^ to equalize the luminance on the same region among different patient images. Images were resized to the scale so that long side length was close to 800 pixels while maintaining the aspect ratio. Augmentations were employed during training, including scale and aspect ratio floating, image shifting, pixel noise, brightness floating, 360-degree random rotation, random flip, and random color inverse.

### Model architecture

**Figure 5:**
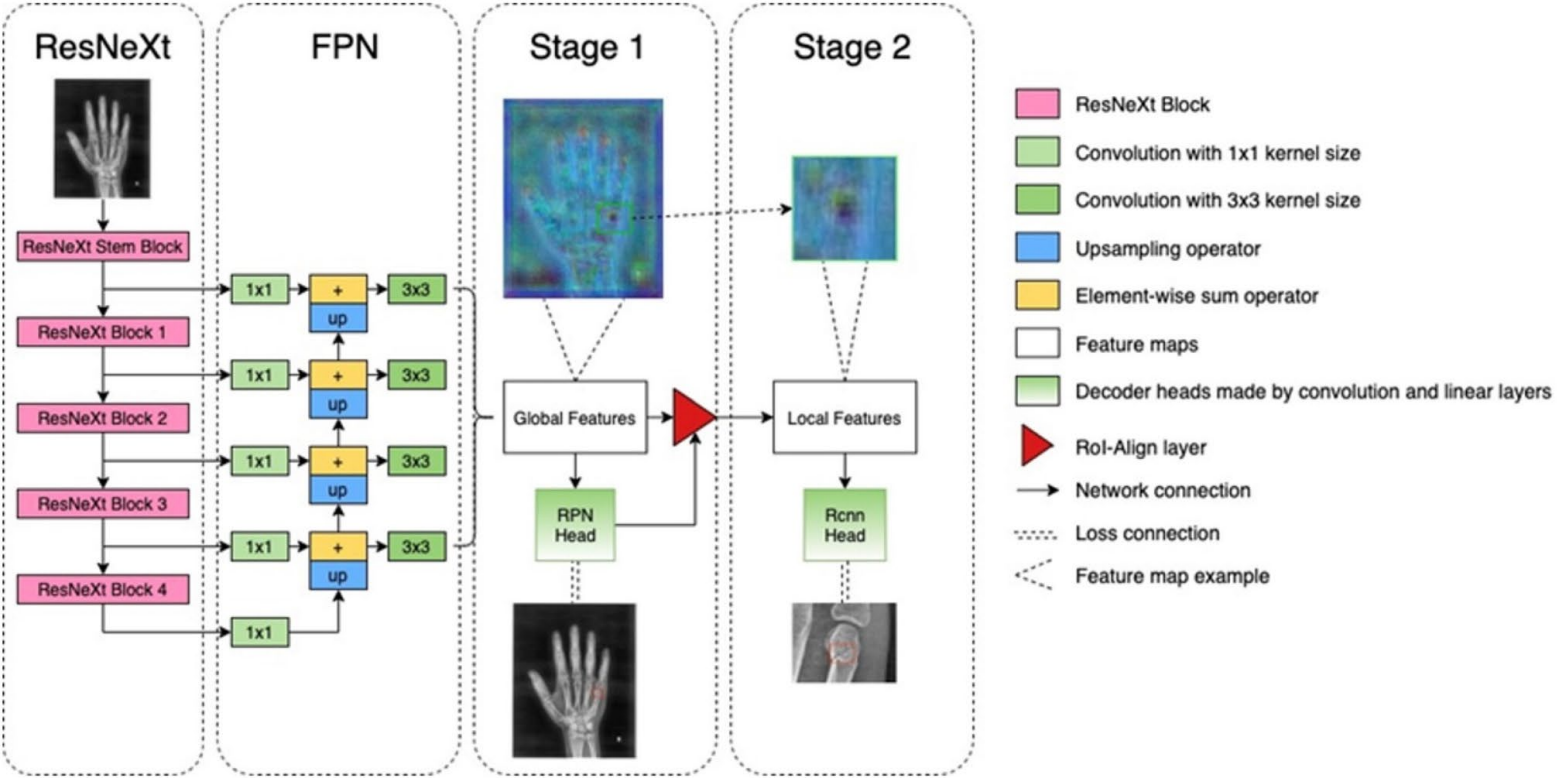
Object detection architecture on fractures dataset.

The artificial neural network learning model was built on the ResNeXt+FPN architecture, which was the state of the art in the object domain (Fig. 5). Pre-processed radiographs were firstly sent to the initial encoder (ResNeXt). Then, a feature pyramid network (FPN) collected the feature maps in different scales produced by ResNeXt, and fused them to generate features with both definition and semantic information. The outputs of FPN, referred to as global feature maps, were forwarded by RPN (region proposal decoder)-Head CNN (Convolution Neural Network)-decoder to make global box predictions to fit the labels of lesions on the whole image. Next, the global features were cropped by RoI-Align^12^ operator according to the global box predictions, and the whole image was cropped simultaneously by the same boxes to make corresponding local labels. Local features were forwarded by Region-based Convolution Neural Network (RCNN)-Head decoder to fit local box labels. Finally, at the clinic scene, RCNN-Head decoder would pick fracture lesion from radiographs instance-by-instance.

The whole structure is essentially an encoder-decoder structure but connected to a so called RoI-Align operator. The input of the network is one radiograph and its label. The radiograph was fed into the CNN encoder, and the ResNext+FPN^9,10^ encoder which is an enhanced version of a typical ResNet encoder was used. The ResNext was made by stacking several identical ResBlocks which are a serialized connection of the convolution, group convolution, non-linear activation function, batch normalization layer, and down-sampling layer. Because each ResBlock ends with a down-sampling layer, the final feature map output will be down-sampled by 4 × 2 n times, where n represents the number of ResBlocks stacked. The output of ResNext has a low definition, such that we cannot localize fracture lesions precisely if using the output feature directly. As such, the FPN (Feature Pyramid Network) was employed to handle this problem. The FPN extracts output feature maps from intermediate ResBlock, concatenates lower definition features with higher definition features by up-sampling the lower one, and adds them together element-wise. Such fusion approach gathers the high level global and the low level local semantic information to harvest the accurate instance prediction. Also, the output of FPN is multi-scale, which makes the network sensible to the objects of different sizes. There is a Region-Proposal decoder supervised and trained by bounding box labels which enclose the rectangle area of fracture lesion; this decoder inferences a Region of Interest (RoI) for each pixel on the FPN output feature maps. The RoI-Align operator then crops on the feature maps according to the RoIs and resizes them into a fixed size, and the RoI features with the highest score predicted by region proposal decoder (RPN Head) would be fed to the next classification and regression decoder (RCNN Head). The classification decoder aims to judge the box prediction to fit the lesion enough or not while the segmentation decoder is designed to refine box prediction.

### FAMO

**Figure 6:**
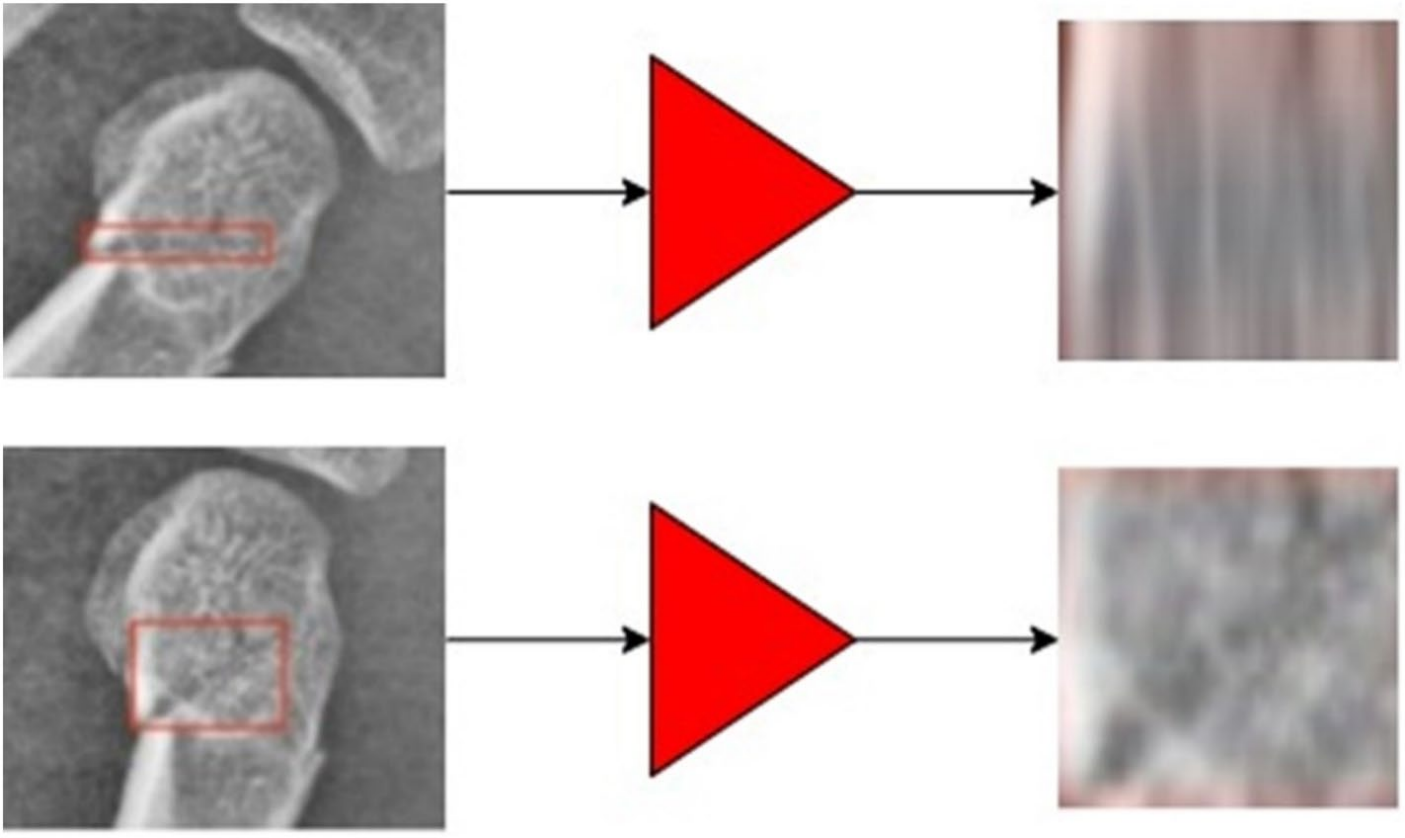
Feature ambiguity in fracture detection. When the fracture line was lying horizontally (upper left), a narrow rectangle box was annotated, and when the fracture line was in an oblique direction, a broader box was annotated (lower left). Both boxes were fed into the RoI-Align operator and resized to the same square shape (right). Processing with the RoI-Align operator caused ambiguity of the image and downgrade the model performance.

In object detection pipelines, the RoI-Align operator is the bridge connecting the global feature and local fracture feature. It is supposed to crop regions proposed by the RPN on the global feature and resize them to unitive square local feature. This operation should be on the assumption that shape deforming would not influence classification. However, in fracture detection, it causes feature ambiguity, which is demonstrated in Fig. 6.

**Figure 7:**
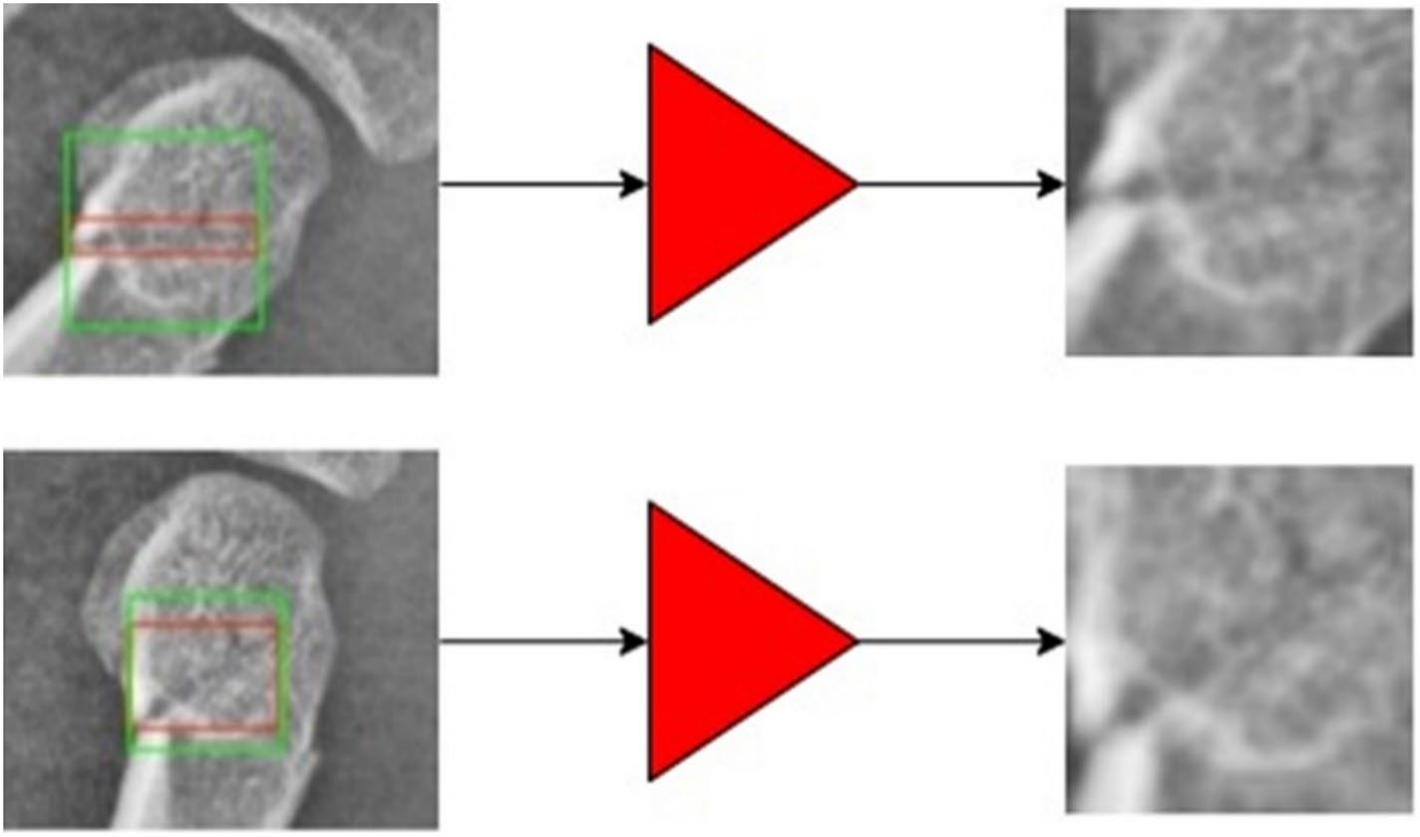
Feature ambiguity in fracture detection was countered in the Feature Ambiguity Mitigate Operator (FAMO) method. On the left, the annotated box was adjusted by expanding its short side (upper left) to the same length as its long side and making a square box (lower left). Thus, the image was not distorted nor ambiguous.

Fractures can be in various directions (Fig. 6). When the fracture line was lying horizontally, a narrow rectangle box was annotated, and when the fracture line was in an oblique direction, a broader box was annotated (left side). Both boxes were fed into the RoI-Align operator and resized to the same square shape (right ones). They were originally the same fractures but were totally different after processed with the RoI-Align operator. The neural-network was forced to classify those ambiguous features into the same class, which would downgrade the model performance. In order to counter this ambiguity in imaging features, the FAMO method was applied (Fig. 7).

The operator adjusts the annotated box by expanding its short side to the same length as its long side and making a square box (green boxes on the left side in Fig. 7). As shown in Fig. 7, after FAMO adjustment, the RoI-Align operator now can crop features without distortion. The model can easily and correctly classify two pictures into the same class.

### Training details

Four NVIDIA 2080Ti GPUs were used. Each GPU could run 1 image synchronously within a training iteration. The training was started with the stochastic gradient descent (SGD) optimizer of linear warming-up learning rate from 0.001 to 0.005 through 500 iterations. According to the linear scaling rule^13^, the learning rate would maintain at 0.005. The model training stopped at the 35th epoch, and we would pick a best epoch among all checkpoints by evaluating them on a validation set.

### Model evaluation

The model was evaluated in three levels: per-case, per-image, and per-fracture level. For per-image level and per-case level, the same metric was used as that used by Thian et al.^7^, where the per-image true positive level requires one box true positive, and the per-case true positive requires one box true positive on any one projection view. For the per-fracture level, the specificity is not testable, because “negative boxes” prevailing in the image which caused specificity for the per-fracture level were always greater than 99%. So instead of using specificity, the true positive was treated as numerator and the sum of true positive and false positive as the denominator to measure in what portion that model predictions were actually fracture.

$${\text{Precision}} = { }\frac{True\,Positive}{{True\,Positive + False\,Positive}}$$

The Average Precision (AP) metric is widely used in object detection research. It averages the precisions at different recall levels, ranging from 0.0 to 1.0. To harvest various precision-recall pairs, different thresholds were set to the box score output by the model, where lower threshold harvested higher recall and higher threshold harvested lower recall.

### Statistical analysis

The statistical analysis was performed with the SPSS 20.0 (IBM, Chicago, IL, USA). The sensitivity, specificity, and AUC in the receiver operator characteristics curve was analyzed. The significant P was set at < 0.05.
